# Ki-67, 21-Gene Recurrence Score, Endocrine Resistance, and Survival in Patients With Breast Cancer

**DOI:** 10.1001/jamanetworkopen.2023.30961

**Published:** 2023-08-30

**Authors:** Janghee Lee, Young-jin Lee, Soong June Bae, Seung Ho Baek, Yoowon Kook, Yoon Jin Cha, Jong Won Lee, Byung Ho Son, Sei Hyun Ahn, Hee Jin Lee, Gyungyub Gong, Joon Jeong, Sae Byul Lee, Sung Gwe Ahn

**Affiliations:** 1 Department of Surgery, Dongtan Sacred Heart Hospital, Hallym University, Dongtan, Republic of Korea; 2 Department of Medicine, Yonsei University Graduate School, Seoul, Republic of Korea; 3 Department of Surgery, Asan Medical Center, University of Ulsan College of Medicine, Seoul, Republic of Korea; 4 Department of Surgery, Gangnam Severance Hospital, Yonsei University College of Medicine, Seoul, Republic of Korea; 5 Institute for Breast Cancer Precision Medicine, Yonsei University College of Medicine, Seoul, Republic of Korea; 6 Department of Pathology, Gangnam Severance Hospital, Yonsei University College of Medicine, Seoul, Republic of Korea; 7 Department of Pathology, Asan Medical Center, University of Ulsan College of Medicine, Seoul, Republic of Korea

## Abstract

**Importance:**

Both high 21-gene recurrence score (RS) and high Ki-67 level are poor prognostic factors in patients with estrogen receptor (ER)–positive *ERBB2*-negative (ER+/*ERBB−*) breast cancer; however, a discrepancy between the 2 has been noted. Survival differences according to these 2 biomarkers are not well known.

**Objective:**

To assess the associations between RS and Ki-67 expression and between Ki-67 expression and recurrence-free survival in patients with ER+/*ERBB−* breast cancer with low RS.

**Design, Setting, and Participants:**

This cohort study included women treated for ER+/*ERBB2*− breast cancer who underwent the 21-gene RS test from March 2010 to December 2020 in 2 hospitals in Korea.

**Exposures:**

Recurrence score and Ki-67 level.

**Main Outcomes and Measures:**

A Cox proportional hazards regression model was used to examine the association of Ki-67 with recurrence-free survival (RFS), while a binary logistic regression model was used to examine the association between Ki-67 and secondary endocrine resistance. High Ki-67 expression was defined as 20% or greater, and low genomic risk as an RS of 25 or less. Secondary endocrine resistance was defined as breast cancer recurrence that occurred after at least 2 years of endocrine therapy and during or within the first year after completing 5 years of adjuvant endocrine therapy.

**Results:**

A total of 2295 female patients were included (mean [SD] age, 49.8 [9.3] years), of whom 1948 (84.9%) were in the low genomic risk group and 1425 (62.1%) had low Ki-67 level. The median follow-up period was 40 months (range, 0-140 months). The RS and Ki-67 level had a moderate correlation (*R* = 0.455; *P* < .001). Of the patients with low Ki-67 level, 1341 (94.1%) had low RS, whereas 607 of 870 patients with high Ki-67 level (69.8%) had low RS. In patients with low RS, the RFS differed significantly according to Ki-67 level (low Ki-67, 98.5% vs high Ki-67, 96.5%; *P* = .002). Among the 1807 patients with low genomic risk who did not receive chemotherapy, high Ki-67 level was independently associated with recurrence (hazard ratio, 2.51; 95% CI, 1.27-4.96; *P* = .008). Recurrence after 3 years differed significantly according to Ki-67 level (low Ki-67, 98.7% vs high Ki-67, 95.7%; *P* = .003), whereas recurrence within 3 years did not differ (low Ki-67, 99.3% vs high Ki-67, 99.3%; *P* = .90). In addition, Ki-67 was associated with secondary endocrine resistance in patients with low RS who did not receive chemotherapy (odds ratio, 2.49; 95% CI, 1.13-5.50; *P* = .02).

**Conclusions and Relevance:**

In this cohort study of patients with ER+/*ERBB2*− breast cancer, a moderate correlation was observed between Ki-67 and RS, and high Ki-67 level in patients with low genomic risk was associated with increased risk of secondary endocrine resistance.

## Key Points

**Question:**

Is Ki-67 expression associated with the 21-gene recurrence score (RS) and with outcomes in patients with breast cancer with a low RS?

**Findings:**

In this cohort study of 2295 patients with breast cancer, a moderate correlation was observed between Ki-67 and RS. Ki-67 had a significant association with disease recurrence beyond 3 years and with secondary endocrine resistance in patients with a low RS.

**Meaning:**

The findings suggest that high Ki-67 level in patients with low genomic risk is associated with increased risk of secondary endocrine resistance.

## Teaser

This cohort study of patients with breast cancer investigates whether Ki-67 expression correlates with the 21-gene recurrence score (RS) and whether high Ki-67 level in patients with a low RS is associated with recurrence and secondary endocrine resistance.

## Introduction

Ki-67 is a nuclear protein associated with proliferation of tumor cells known as a prognostic factor for breast cancer.^1,2,3^ In estrogen receptor (ER)–positive *ERBB2* (formerly *HER2/neu*)–negative (ER+/*ERBB2*−) breast cancer, a higher Ki-67 index is associated with a more aggressive tumor and a higher risk of recurrence.^4^ Recently, Ki-67 has gained attention as a significant biomarker in early ER+/*ERBB2*− breast cancer, particularly after the positive results of the monarchE trial, which demonstrated the benefits of abemaciclib, a CDK4/6 inhibitor, in the adjuvant setting.^5^ Currently, Ki-67 with a cutoff of 20% by MIB-1 pharmDx assay has obtained approvals from the US Food and Drug Administration as a companion diagnostic for adjuvant abemaciclib in high-risk ER+/*ERBB2−* breast cancer.^6,7^

The 21-gene assay is a test that analyzes the activity of 16 cancer-related genes and 5 reference genes in breast cancer tissue to predict the likelihood of recurrence and the potential benefit of chemotherapy.^8,9^ The landmark trials of the 21-gene assay, TAILORx and RxPONDER, have proved that decisions about adjuvant chemotherapy can be made based on the recurrence score (RS), and current guidelines recommend that the 21-gene RS be performed if indicated for early ER+/*ERBB2−* breast cancer.^10,11,12^ As a result, a majority of patients having low genomic risk by 21-gene assay omit adjuvant chemotherapy.

While there is a moderate to strong correlation between Ki-67 and RS,^13,14,15^ they are not interchangeable. Although high Ki-67 levels are associated with increased likelihood of a high RS by the 21-gene assay, Ki-67 alone cannot be used to determine RS.^16^ Generally, in patients with low RS and high Ki-67 level, chemotherapy might be omitted following guidelines endorsing RS as a final determinant despite a disagreement between the 2 biomarkers.^12,17^

The purpose of our study was to investigate the association between the RS and Ki-67 index in a cohort of patients who received adjuvant treatment based on the results of the 21-gene assay. Specifically, we aimed to assess the agreement between high Ki-67 and high RS and to evaluate outcomes in patients with high Ki-67 and low RS.

## Methods

### Study Population

This cohort study followed the Strengthening the Reporting of Observational Studies in Epidemiology (STROBE) reporting guideline for observational studies. Data from patients who underwent curative surgery for ER+/*ERBB2−* invasive breast cancer were retrospectively collected at Gangnam Severance Hospital and Asan Medical Center from March 2010 to December 2020. These patients had primary breast cancer with no evidence of metastasis to other organs at the time of diagnosis. Since the 21-gene RS test (Oncotype DX) became clinically available, both hospitals have been conducting the test only on patients who met the indications at that time and agreed to undergo the test.^18^ Ultimately, we selected patients who had an available 21-gene RS assay result.^19^ Clinical information on patients and pathological data on breast cancer were obtained through a comprehensive review of medical records. Patients who had received neoadjuvant chemotherapy were excluded from the study since they did not undergo 21-gene RS testing. The process for patient selection is depicted in the diagram provided in eFigure 1 in Supplement 1. The study protocol received approval from the institutional review boards of Gangnam Severance Hospital and Asan Medical Center. Due to the retrospective nature of the study, the requirement for informed consent was waived by the institutional review boards.

### Ki-67 Index

The Ki-67 index was measured centrally in untreated breast tumor tissue from surgical specimens. An immunohistochemistry assay was performed on formalin-fixed paraffin-embedded (FFPE) tissue using MIB-1, anti–Ki-67 antibody. The interpretation of the results was carried out by expert pathologists (Y.J.C., H.J.L., and G.Y.G.) using a light microscope. Ki-67 expression was reported as the percentage (ranging from 0% to 100%) of tumor cells showing positive staining. In cases in which the Ki-67 result was provided as a range, such as 15% to 30%, the median value of the range was used. High Ki-67 expression was defined using a cutoff value of 20%.^5,20^

### 21-Gene RS Assay

We performed a 21-gene RS assay on tumor tissue from surgical specimens of all enrolled patients by commissioning a central laboratory of Genomic Health. The 21-gene RS assay quantified expression of 21 genes in FFPE tissue using high-throughput, real-time reverse transcription–polymerase chain reaction.^8^ The panel of genes consisted of 16 cancer-related genes including proliferative genetic biomarkers, such as *MKI-67*, *STK15*, *BIRC5*, *CCNB1*, and *MYBL2*, and 5 reference genes. In case of multiple tumors, the RS test was performed on the 1 or 2 largest tumors. In addition, when the RS test was performed on more than 1 tumor sample, a higher score was adopted as the RS result. Patients who failed the 21-gene RS test were excluded.

Initially, the 21-gene RS test was validated by dividing results into low (RS, <18), intermediate (RS, 18-30), and high (RS, >31) risk.^9^ However, in the TAILORx trial, node-negative patients with an RS of more than 25 were classified as a high-risk group.^10^ Moreover, an RS of 25 was set as the high-risk criterion in the RxPONDER study, which evaluated the performance of the 21-gene RS assay in node-positive patients.^11^ Based on these trials, our study classified patients into low and high genomic risk groups with an RS cutoff value of 25.

### Secondary Endocrine Resistance

Secondary endocrine resistance refers to the development of acquired resistance resulting from the use of antiestrogen agents such as tamoxifen and aromatase inhibitors. In the context of early breast cancer, secondary endocrine resistance is clinically defined as the recurrence of cancer that takes place after a minimum of 2 years of endocrine therapy and either during or within the first year following completion of 5 years of adjuvant endocrine therapy. In our study, we focused on patients classified as having a low RS who did not undergo chemotherapy and exhibited secondary endocrine resistance. Additional information regarding the patients with secondary endocrine resistance can be found in eTable 1 in Supplement 1.

### Statistical Analysis

The primary objective of our study was to compare recurrence-free survival (RFS) according to Ki-67 expression in groups classified as having genomic risk. The RFS was defined as the period between breast cancer surgery and recurrence of breast cancer. Recurrence included locoregional recurrence in the ipsilateral breast or regional lymph node and distant recurrence. Contralateral breast cancer was excluded as a recurrence event because we regarded it as a secondary malignant neoplasm. We used Kaplan-Meier survival curves for RFS analysis, and factors associated with RFS were identified using both univariate and multivariate Cox proportional hazards regression models. Furthermore, to examine the association between Ki-67 and recurrence with secondary endocrine resistance, we used a binary logistic regression model.

The correlation between the continuous Ki-67 index and 21-gene RS was evaluated using Pearson correlation coefficient. The analytic performance of Ki-67 for 21-gene RS was assessed using the area under the receiver operating characteristic (ROC) curve (AUC). Differences between groups were analyzed using the χ^2^ test for categorical variables and 1-way analysis of variance for continuous variables, with confirmation by the Levene test for equality of variances. All statistical tests were 2-sided, and *P* < .05 was considered to be statistically significant. Statistical analyses were conducted using R, version 3.6.1 (R Project for Statistical Computing) and GraphPad Prism, version 9 (GraphPad Software).

## Results

### Agreement Between Ki-67 and RS

Of 14 923 female patients who underwent curative surgery for ER+/*ERBB2−* invasive breast cancer, a total of 2295 were included in the study (mean [SD] age, 49.8 [9.3] years); among them, 1948 (84.9%) belonged to the low genomic risk group and 1425 (62.1%) had low Ki-67 expression. The baseline characteristics of the patients are summarized in Table 1. Among the included patients, 1486 (64.7%) were premenopausal, 1481 (64.5%) had T1 tumors, and 1862 (81.1%) had node-negative disease. Progesterone receptor negativity was observed in 296 patients (12.9%), while grade 3 tumors were present in 225 (9.8%). The rate of lymphovascular invasion was 24.9% (571 of 2295). Breast-conserving surgery was performed in 1726 patients (75.2%), and 1854 (80.8%) did not receive adjuvant chemotherapy. Ovarian function suppression was added to endocrine therapy in 561 cases (24.4%).

**Table 1.** Baseline Characteristics of Enrolled Patients

Characteristic	Enrolled patients (N = 2295)^a^
Age, mean (SD), y	49.8 (9.3)
Menopausal status	
Premenopausal	1486 (64.7)
Postmenopausal	809 (35.3)
21-Gene RS	
Median (range)	17.9 (0.0-72.0)
≤25	1948 (84.9)
>25	347 (15.1)
Ki-67, %	
Median (range)	15.1 (0.0-90.0)
<20	1425 (62.1)
≥20	870 (37.9)
Tumor size, mm	
≤2	1481 (64.5)
>2	814 (35.5)
Metastatic LN	
Negative	1862 (81.1)
Positive	433 (18.9)
PR	
Negative	296 (12.9)
Positive	1999 (87.1)
*ERBB2*	
Negative	944 (41.1)
Low positive	1351 (58.9)
HG	
I or II	2064 (89.9)
III	225 (9.8)
Unknown	6 (0.3)
LVI	
Negative	1724 (75.1)
Positive	571 (24.9)
Breast surgery	
BCS	1726 (75.2)
Mastectomy	569 (24.8)
Chemotherapy	
Not performed	1854 (80.8)
Performed	436 (19.0)
Unknown	5 (0.2)
Endocrine therapy	
Not performed	26 (1.1)
SERM	1513 (65.9)
AI	748 (32.6)
Other or unknown	8 (0.3)
OFS	
Not performed	1732 (75.5)
Performed	561 (24.4)
Unknown	2 (0.1)
Radiotherapy	
Not performed	532 (23.2)
Performed	1755 (76.5)
Unknown	8 (0.3)

Abbreviations: BCS, breast conserving surgery; HG, histologic grade; LN, lymph node; LVI, lymphovascular invasion; OFS, ovarian function suppression; PR, progesterone receptor; RS, recurrence score; SERM, selective estrogen receptor modulator.

^a^ Data are presented as the number (percentage) of patients unless otherwise indicated.

**Figure 1.**
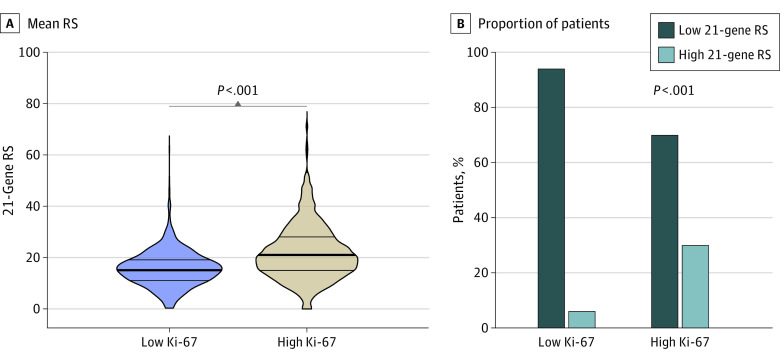
Comparisons Between 21-Gene Recurrence Score (RS) and Ki-67 Level Student *t* test (A) and χ^2^ test (B) were used to calculate *P* values.

We first examined the correlation between RS and Ki-67 and found a moderate correlation (*R* = 0.455; *P* < .001) (eFigure 2 in Supplement 1). The ROC curve analysis showed that Ki-67 had an AUC of 0.79 (95% CI, 0.76-0.81) for high RS (eFigure 2 in Supplement 1). Moreover, the mean RS was significantly higher in the high Ki-67 group compared with the low Ki-67 group (21.9 [95% CI, 21.20-22.60] vs 15.5 [95% CI, 15.13-15.85]; *P* < .001) (Figure 1A).

When we investigated the agreement between the 2 biomarkers (Figure 1B), an agreement was noted in 1604 patients (69.9%), while a disagreement was observed in 691 (30.1%). Of the 1425 patients with low Ki-67 level, 1341 (94.1%) had low RS. However, among the 870 patients with high Ki-67 level (37.9%), a majority (607 [69.8%]) had low RS. High Ki-67 was observed in 607 of 1948 patients (31.2%) classified as having low RS.

### RFS According to RS and Ki-67

At a median follow-up of 40 months (range, 1-140 months), the 5-year RFS was 98.2%. A total of 65 recurrence events, including 40 cases of distant metastases, were observed. Recurrence-free survival differed significantly according to RS (97.9% for low RS vs 93.1% for high RS; *P* < .001) and Ki-67 (98.3% for low Ki-67 vs 95.3% for high Ki-67; *P* < .001) (eFigure 3 in Supplement 1).

We then examined the association of Ki-67 with RFS in 2 groups stratified by RS. Within the group with low RS, high Ki-67 level was significantly associated with inferior RFS (low Ki-67, 98.5% vs high Ki-67, 96.5%; *P* = .002) (eFigure 4 in Supplement 1). However, Ki-67 was not found to be associated with the rate of recurrence in the group with high RS (low Ki-67, 95.2% vs high Ki-67, 92.4%; *P* = .27) (eFigure 4 in Supplement 1). In the multivariate analysis, high Ki-67 remained associated with RFS in patients with low RS (hazard ratio [HR], 2.26; 95% CI, 1.19-4.29; *P* = .01) (eTable 2 in Supplement 1). Histologic grade (HG) was identified as another factor associated with RFS.

### Secondary Endocrine Resistance by High Ki-67 Level in Patients With Low RS

**Figure 2.**
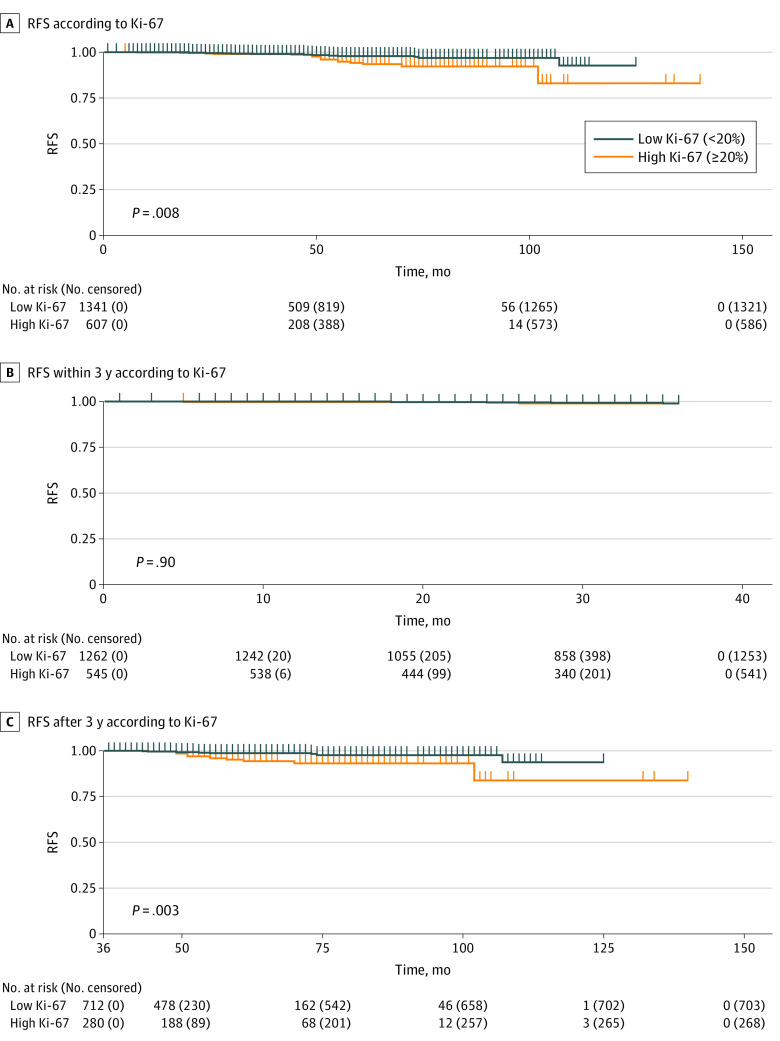
Recurrence-Free Survival (RFS) Analysis in Patients With Low Recurrence Score Who Did Not Receive Chemotherapy *P* values are from the log-rank test.

Despite the RS-guided adjuvant treatments received by the enrolled patients, a small proportion of patients with low RS (137 of 1948 [7.0%]) underwent chemotherapy based on other clinical and pathological considerations. We identified 1807 patients with low RS who did not receive chemotherapy (92.8%) and summarized the clinicopathologic features and treatment modalities in eTable 3 in Supplement 1. Of the 607 patients with low RS and high Ki-67, 545 (89.8%) did not receive adjuvant chemotherapy; in these patients, high Ki-67 remained associated with RFS (low Ki-67, 98.6% vs high Ki-67, 96.9%; *P* = .008) (Figure 2A). In the multivariate analysis, Ki-67 was identified as an independent factor associated with recurrence (HR, 2.51; 95% CI, 1.27-4.96; *P* = .008), along with HG (eTable 4 in Supplement 1).

We observed a divergence in the RFS curve based on Ki-67 status after 3 years. Within the initial 3 years, there was no significant difference in RFS based on Ki-67 (low Ki-67, 99.3% vs high Ki-67, 99.3%; *P* = .90) (Figure 2B). However, high Ki-67 level was associated with an increased risk of recurrence after 3 years among patients who had remained recurrence free during the first 3 years (low Ki-67, 98.7% vs high Ki-67, 95.7%; *P* = .003) (Figure 2C). In the multivariate analysis, Ki-67 was associated with RFS beyond 3 years (HR, 4.19; 95% CI, 1.57-11.22; *P* = .004) (Table 2). Conversely, only high HG was associated with recurrence within 3 years (HR, 5.50; 95% CI, 1.48-20.38; *P* = .01).

**Table 2.** Multivariate Analysis of Factors Associated With RFS According to Period of Recurrence in Patients With Low Genomic Risk Who Did Not Receive Chemotherapy

Factor	RFS within 3 y		RFS after 3 y	
	HR (95% CI)	*P* value	HR (95% CI)	*P* value
Menopausal status				
Premenopausal	1 [Reference]^a^	.53	1 [Reference]	.87
Postmenopausal	0.63 (0.15-2.70)		1.10 (0.35-3.46)	
Tumor size, mm				
≤20	1 [Reference]	.87	1 [Reference]	.52
>20	1.10 (0.35-3.53)		0.73 (0.27-1.95)	
Metastatic LN				
Negative	1 [Reference]	.86	1 [Reference]	.81
Positive	1.12 (0.31-3.99)		1.14 (0.41-3.12)	
PR				
Negative	1 [Reference]	.92	1 [Reference]	.07
Positive	0.90 (0.11-7.64)		0.34 (0.11-1.07)	
*ERBB2*				
Negative	1 [Reference]	.71	1 [Reference]	.37
Low positive	1.24 (0.40-3.86)		0.67 (0.28-1.61)	
HG				
I or II	1 [Reference]	.01	1 [Reference]	.07
III	5.50 (1.48-20.38)		2.94 (0.92-9.39)	
LVI				
Negative	1 [Reference]	.11	1 [Reference]	.89
Positive	2.64 (0.81-8.66)		1.08 (0.40-2.92)	
Ki-67, %				
<20	1 [Reference]	.99	1 [Reference]	.004
≥20	0.99 (0.28-3.45)		4.19 (1.57-11.22)	
Breast surgery				
BCS	1 [Reference]	.85	1 [Reference]	.26
Mastectomy	0.66 (0.01-50.80)		3.30 (0.41-26.46)	
OFS				
Not performed	1 [Reference]	.76	1 [Reference]	.89
Performed	0.81 (0.21-3.10)		0.92 (0.30-2.88)	
Radiotherapy				
Not performed	1 [Reference]	.37	1 [Reference]	.97
Performed	0.13 (0.00-10.31)		1.04 (0.13-8.19)	

Abbreviations: BCS, breast conserving surgery; HG, histologic grade; HR, hazard ratio; LN, lymph node; LVI, lymphovascular invasion; OFS, ovarian function suppression; PR, progesterone receptor; RFS, recurrence-free survival.

Furthermore, we evaluated the clinical value of Ki-67 in terms of secondary endocrine resistance. In the multivariate analysis using a binary logistic regression model, among 26 patients who did not undergo chemotherapy and exhibited secondary endocrine resistance, Ki-67 was found to be associated with secondary endocrine resistance (odds ratio, 2.49; 95% CI, 1.13-5.50; *P* = .02) (Table 3).

**Table 3.** Univariate and Multivariate Analysis of Factors Associated With Secondary Endocrine Resistance in Patients With Low Genomic Risk Who Did Not Receive Chemotherapy

Factor	Univariate analysis		Multivariate analysis	
	OR (95% CI)	*P* value	OR (95% CI)	*P* value
Menopausal status				
Premenopausal	1 [Reference]	.56	NA	NA
Postmenopausal	1.27 (0.27-2.83)		NA	NA
Tumor size, mm				
≤20	1 [Reference]	.40	NA	NA
>20	1.40 (0.64-3.07)		NA	NA
Metastatic LN				
Negative	1 [Reference]	.16	NA	NA
Positive	1.79 (0.79-4.06)		NA	NA
PR				
Negative	1 [Reference]	.06	NA	NA
Positive	0.39 (0.14-1.04)		NA	NA
*ERBB2*				
Negative	1 [Reference]	.39	NA	NA
Low positive	0.71 (0.33-1.55)		NA	NA
HG				
I or II	1 [Reference]	.03	1 [Reference]	.05
III	3.47 (1.16-10.38)		3.13 (1.02-9.58)	
LVI				
Negative	1 [Reference]	.23	NA	NA
Positive	1.65 (0.73-3.73)		NA	NA
Ki-67, %				
<20	1 [Reference]	.02	1 [Reference]	.02
≥20	2.50 (1.15-5.43)		2.49 (1.13-5.50)	
Breast surgery				
BCS	1 [Reference]	.003	1 [Reference]	.32
Mastectomy	3.24 (1.49-7.08)		2.77 (0.37-20.53)	
OFS				
Not performed	1 [Reference]	.54	NA	NA
Performed	1.36 (0.56-3.03)		NA	NA
Radiotherapy				
Not performed	1 [Reference]	.004	1 [Reference]	.79
Performed	0.32 (0.15-0.70)		0.76 (0.10-5.67)	

Abbreviations: BCS, breast conserving surgery; HG, histologic grade; LN, lymph node; LVI, lymphovascular invasion; NA, not applicable; OFS, ovarian function suppression; OR, odds ratio; PR, progesterone receptor.

## Discussion

In this study, we found a moderate correlation and a substantial agreement between RS and Ki-67 expression in patients with ER+/*ERBB2−* breast cancer, with an agreement rate of 69.9%. Moreover, we found that high Ki-67 levels were associated with an increased risk of recurrence in the group with low RS without chemotherapy as well as in the entire population with low RS. Additionally, our findings demonstrated that patients with high Ki-67 level had significantly reduced RFS after 3 years from the operation, indicating a potential association between high Ki-67 expression and secondary endocrine resistance in individuals with low RS who experienced relapse after 2 years of adjuvant endocrine therapy.

When calculating RS, the most significant weight is assigned to 5 proliferation genes among the 21 genes included, including MKI, which specifically encodes Ki-67.^10^ However, there remains a notable discrepancy between Ki-67 expression and RS, particularly in patients with low genomic risk. Crager et al^14^ previously reported that approximately 25% of patients with low genomic risk exhibited high Ki-67 expression (≥20%). Our study yielded similar results, with high Ki-67 observed in 31.2% of patients classified as having low RS. Furthermore, another study indicated that 79% of patients with high Ki-67 expression had an RS of 25 or less,^15^ which aligns with our study, in which 69.8% of patients with high Ki-67 expression were classified as having a low RS.

The disparity between RS and Ki-67 may be attributed to the fact that Ki-67 is not the sole determinant of RS, despite its correlation with RS. The RS is determined by a multigene panel that encompasses not only proliferation genes but also genes associated with ER, *ERBB2*, invasion, and other factors.^8^ As a result, pathological factors, such as HG, progesterone receptor status, and lymphovascular invasion, may be associated with RS.^21,22,23^ This could explain the observed differences between RS and Ki-67. Additionally, previous studies have demonstrated that Ki-67 is a robust prognostic factor^24^ and can serve as a marker for predicting the effectiveness of neoadjuvant chemotherapy.^25^ However, it cannot accurately predict the benefit of adjuvant chemotherapy,^26^ and it should not be used interchangeably with RS, due to the high disagreement rates observed between RS and Ki-67 within the group with high Ki-67 level. Consequently, it is reasonable that 545 of 607 patients with low RS and high Ki-67 (89.8%) did not receive adjuvant chemotherapy.

To our knowledge, our study is the first to demonstrate a survival difference based on Ki-67 expression in patients with low genomic risk. Importantly, our findings suggest that this difference was primarily attributed to recurrence after 3 years, known as secondary endocrine resistance, rather than early recurrence within 3 years. Some researchers have put forth the argument that high Ki-67 levels are associated with late recurrence of ER+ tumors and have proposed considering extended endocrine therapy for such cases.^27,28^ Our findings are also in line with these studies. These results imply that alternative approaches, rather than chemotherapy, may be necessary for individuals with low genomic risk and high Ki-67 expression, as they face a risk of relapse after 3 years. Through our study, we shed light on the clinical need for strategies to overcome secondary endocrine resistance in this specific patient subset.

Persistent expression of cyclin D and phosphorylation of retinoblastoma tumor suppressor protein (Rb) has been recognized as 1 of the major causes of endocrine resistance.^29^ CDK4/6 inhibitors are recognized for their ability to reverse endocrine resistance, as they function by inducing G1 cell cycle arrest and inhibiting cellular proliferation through the suppression of Rb phosphorylation.^30,31^ In addition, the monarchE trial demonstrated the efficacy of abemaciclib, 1 of the CDK4/6 inhibitors, in patients with ER+/*ERBB2−* cancer with high Ki-67 expression.^5^ Recently updated findings from the monarchE study have revealed a significant carryover effect that extends for approximately 4 years after the completion of a 2-year abemaciclib treatment regimen.^32^ These findings suggest that the abemaciclib-combined regimen could potentially serve as a valuable approach to overcome secondary endocrine resistance. However, since the majority of the patients in the monarchE trial received chemotherapy,^33^ an additional prospective trial is needed to demonstrate clinical benefit of CDK4/6 inhibitor in patients who do not receive chemotherapy.

### Limitations

A limitation of the study is the retrospective study design, which may introduce potential selection bias. However, we mitigated this concern by gathering data from a large number of patients who underwent the 21-gene test, a centrally controlled test performed in a single laboratory, in conjunction with other clinical and pathological variables. Furthermore, we were unable to assess Ki-67 using the pharmDx assay, which is approved as a companion diagnostic for adjuvant abemaciclib. Additionally, there may have been bias related to interobserver variations in the interpretation of Ki-67 among pathologists due to the challenges involved in standardizing Ki-67.^34,35^ Nonetheless, we used the same MIB-1 antibody as the pharmDx assay and ensured consistent interpretation of Ki-67 immunohistochemistry stain results by experienced pathologists from 2 reputable institutions. The interpretation was conducted following the established protocol outlined by the working group.^36^ A significant portion of the issues related to the standardization of Ki-67 could be resolved in the future through the development and widespread adoption of automated digital image analysis methods.^37,38^

## Conclusions

In this cohort study, a moderate correlation was observed between Ki-67 and RS. In addition, Ki-67 was found to have a significant association with disease recurrence beyond 3 years and with secondary endocrine resistance in patients with a low RS. The findings suggest that there is a need for further studies to assess innovative approaches, such as combined therapy with CDK4/6 inhibitors, for patients with high Ki-67 expression, even if they have a low RS.

## Notes

Supplement 1. **eFigure 1.** CONSORT Diagram of Enrolled Patients **eFigure 2.** Association Between Ki-67 and 21-Gene RS **eFigure 3.** Kaplan-Meier Survival Curve for RFS According to Genomic Risk and Ki-67 **eFigure 4.** RFS Analysis According to Ki-67 in Each Low and High Genomic Risk Group **eTable 1.** Clinical and Recurrence Information of Patients With Secondary Endocrine Resistance **eTable 2.** Univariate and Multivariate Analysis of Factors for RFS in Patients With Low Genomic Risk **eTable 3.** Comparison of Baseline Characteristics According to Ki-67 in Patients With Low Genomic Risk Who Did Not Receive Chemotherapy **eTable 4.** Univariate and Multivariate Analysis of Factors for RFS in Patients With Low Genomic Risk Who Did Not Receive Chemotherapy

Supplement 2.
Data Sharing Statement
