# Supplementary material for: Ki-67, 21-Gene Recurrence Score, Endocrine Resistance, and Survival in Patients With Breast Cancer
Source: JAMA Netw Open. 2023 Aug 30;6(8):e2330961. doi: 10.1001/jamanetworkopen.2023.30961 (PMC10469325; doi:10.1001/jamanetworkopen.2023.30961)
Supplement: Supplement 1. — eFigure 1. CONSORT Diagram of Enrolled Patients eFigure 2. Association Between Ki-67 and 21-Gene RS eFigure 3. Kaplan-Meier Survival Curve for RFS According to Genomic Risk and Ki-67 eFigure 4. RFS Analysis According to Ki-67 in Each Low and High Genomic Risk Group eTable 1. Clinical and Recurrence Information of Patients With Secondary Endocrine Resistance eTable 2. Univariate and Multivariate Analysis of Factors for RFS in Patients With Low Genomic Risk eTable 3. Comparison of Baseline Characteristics According to Ki-67 in Patients With Low Genomic Risk Who Did Not Receive Chemotherapy eTable 4. Univariate and Multivariate Analysis of Factors for RFS in Patients With Low Genomic Risk Who Did Not Receive Chemotherapy [file jamanetwopen-e2330961-s001.pdf]

Supplementary Online Content

Lee J, Lee YJ, Bae SJ, et al. Ki-67, 21-gene recurrence score, endocrine resistance, and survival in patients with breast cancer. *JAMA Netw Open*. 2023;6(8):e2330961. doi:10.1001/jamanetworkopen.2023.30961

eFigure 1. CONSORT Diagram of Enrolled Patients

eFigure 2. Association Between Ki-67 and 21-Gene RS

eFigure 3. Kaplan-Meier Survival Curve for RFS According to Genomic Risk and Ki-67

eFigure 4. RFS Analysis According to Ki-67 in Each Low and High Genomic Risk Group

eTable 1. Clinical and Recurrence Information of Patients With Secondary Endocrine Resistance

eTable 2. Univariate and Multivariate Analysis of Factors for RFS in Patients With Low Genomic Risk

eTable 3. Comparison of Baseline Characteristics According to Ki-67 in Patients With Low Genomic Risk Who Did Not Receive Chemotherapy

eTable 4. Univariate and Multivariate Analysis of Factors Associated With RFS in Patients With Low Genomic Risk Who Did Not Receive Chemotherapy

This supplementary material has been provided by the authors to give readers additional information about their work.

© 2023 Lee J et al. *JAMA Network Open*.

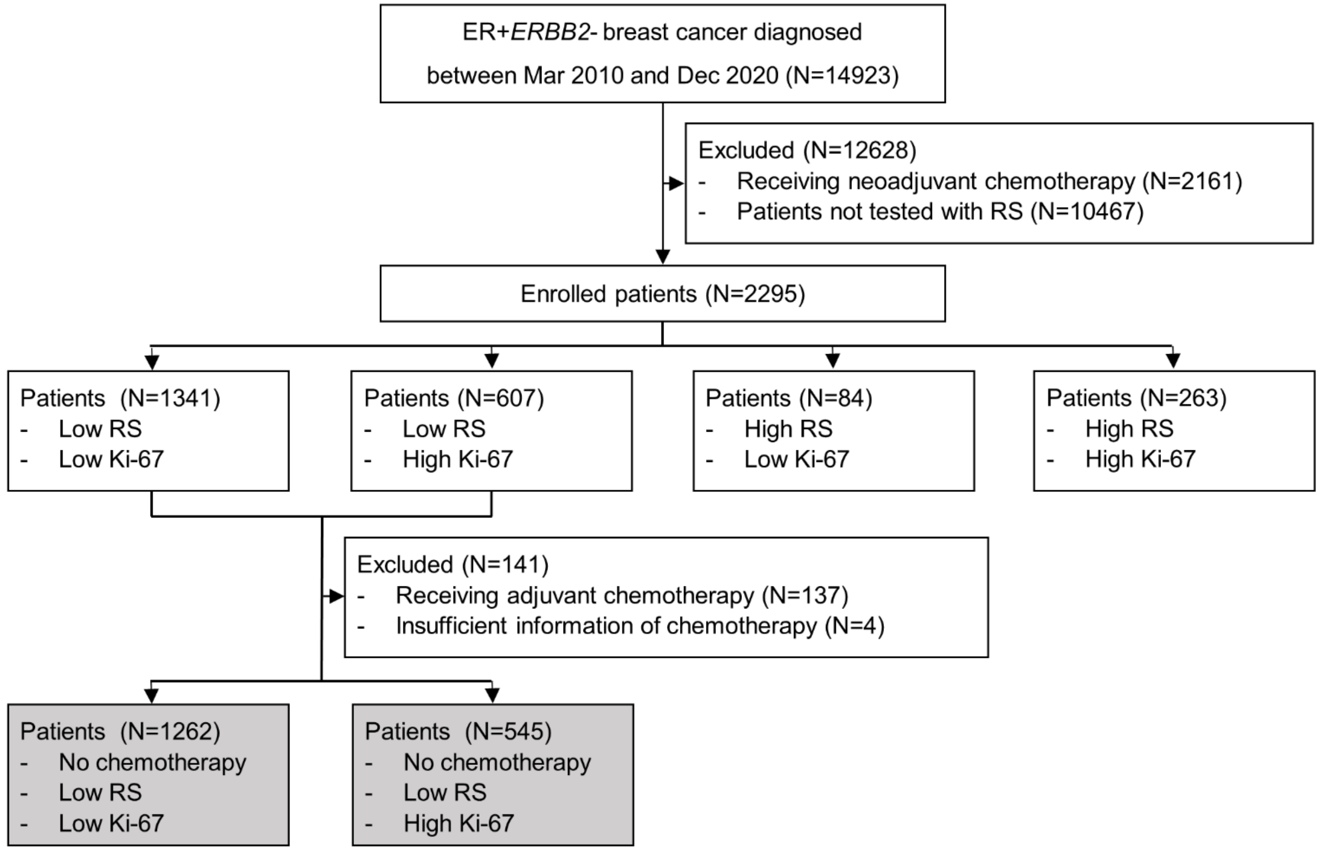

eFigure 1. Consort diagram of enrolled patients
ER, estrogen receptor; RS, recurrence score

© 2023 Lee J et al. *JAMA Network Open*.

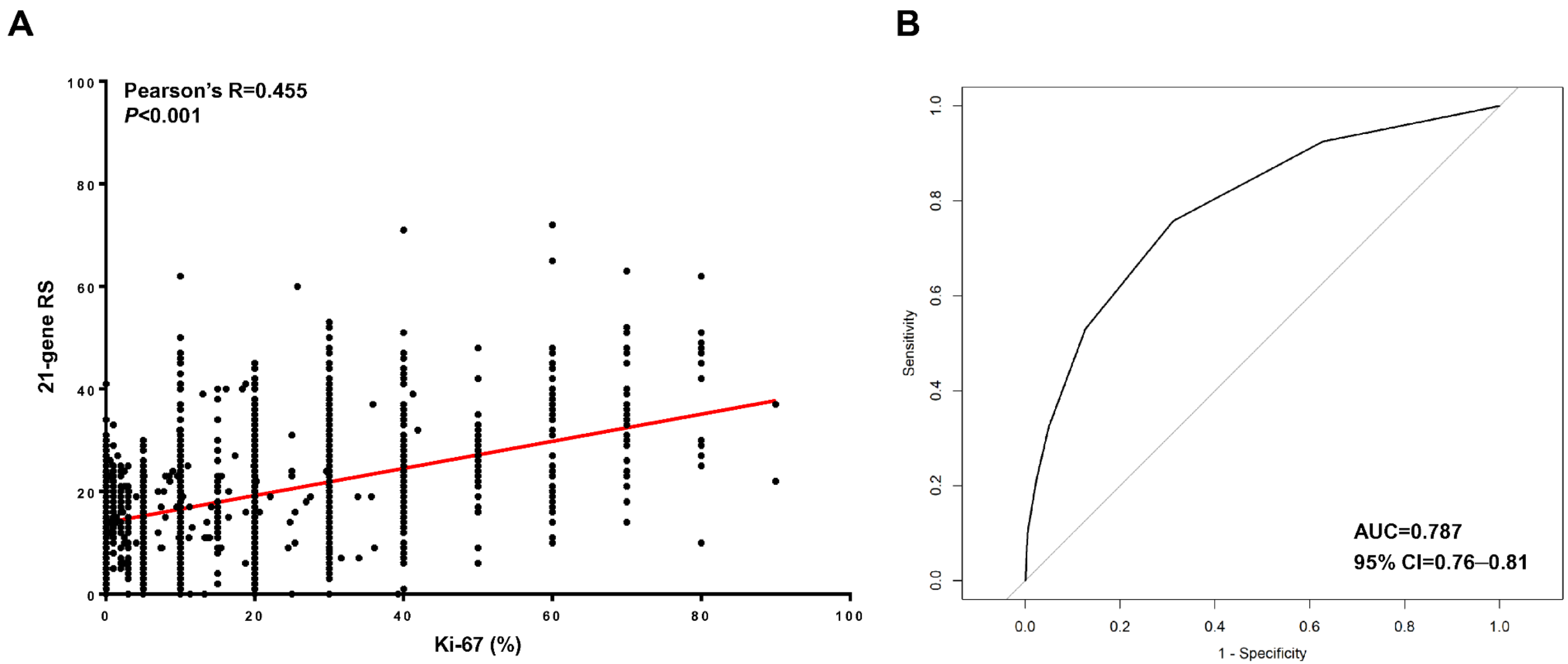

eFigure 2. Association between Ki-67 and 21-gene RS; (A) Pearson correlation analysis between Ki-67 and 21-gene RS (Pearson;s R=0.455; *P*<0.001). (B) ROC curve for prediction of high genomic risk of high Ki-67 (AUC, 0.787, 95% CI, 0.76–0.81)
RS, recurrence score; ROC, receiver operating characteristics; AUC, area under the curve; CI, confidence intervals

© 2023 Lee J et al. *JAMA Network Open*.

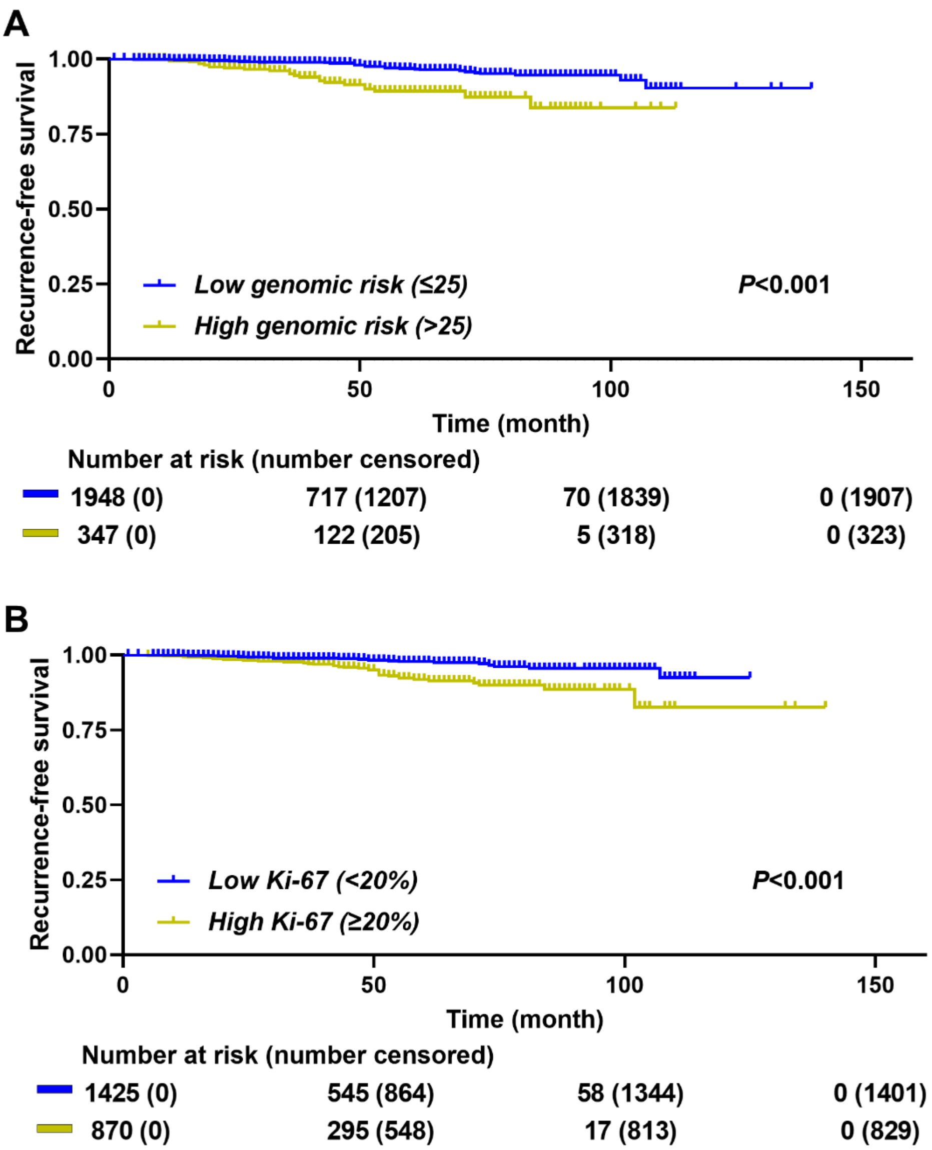

eFigure 3. Kaplan-Meier survival curve for RFS according to genomic risk and Ki-67. (A) Comparison of RFS according to genomic risk (*P*<0.001); (B) Comparison of RFS according to Ki-67 (*P*<0.001)

© 2023 Lee J et al. *JAMA Network Open*.

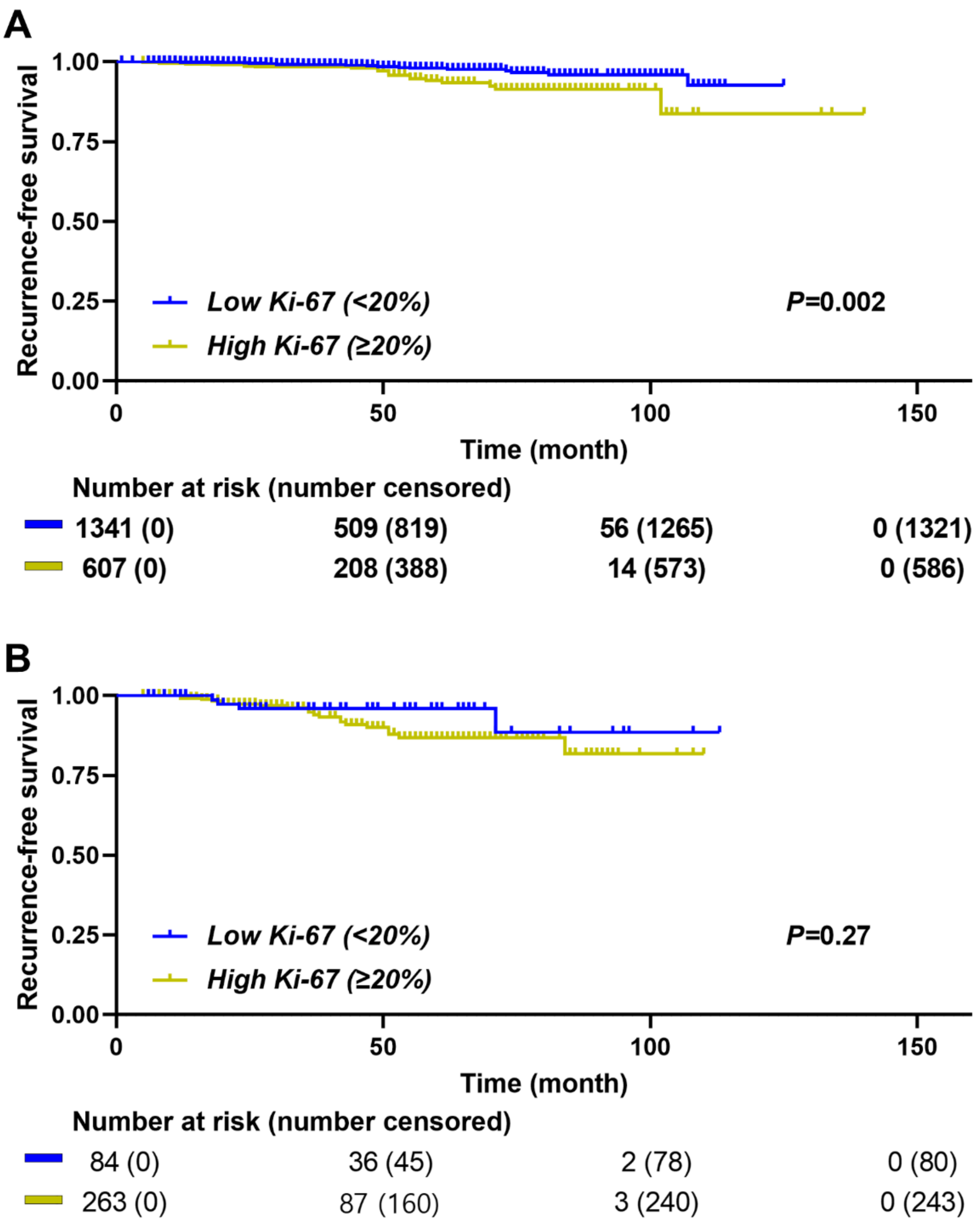

eFigure 4. RFS analysis according to Ki-67 in each low and high genomic risk group. (A) RFS according to Ki-67 in patients with low genomic risk (*P*=0.002); (B) RFS according to Ki-67 in patients with high genomic risk (*P*=0.27)
RFS, recurrence-free survival

© 2023 Lee J et al. *JAMA Network Open*.

eTable 1. Clinical and recurrence information of patients with secondary endocrine resistance

Patient number	Age	Stage	21-gene RS	Ki-67 (%)	Duration from the start of endocrine therapy (month)	Recurrence type
1	57	I	23	30	24	LRR
2	63	I	17	3	24	DR
3	68	I	4	20	25	DR
4	55	II	25	10	27	LRR/DR
5	45	II	16	<1	29	DR
6	41	II	8	<1	29	DR
7	43	I	14	3	34	LRR
8	44	I	19	10	42	LRR/DR
9	40	I	15	30	43	DR
10	43	II	18	10	46	DR
11	66	II	8	10	47	LRR
12	27	II	11	30	48	LRR
13	59	I	25	10	48	DR
14	70	I	7	34	48	LRR
15	52	I	18	30	50	LRR/DR
16	42	II	15	20	50	LRR
17	67	II	24	40	51	LRR
18	49	II	20	10	52	LRR
19	45	I	19	10	53	DR
20	47	II	18	30	54	DR
21	58	II	21	20	54	LRR
22	49	II	15	20	57	DR
23	34	I	21	20	60	DR
24	37	I	21	40	69	LRR

© 2023 Lee J et al. *JAMA Network Open*.

25	44	I	18	15	72	LRR
26	51	I	17	10	73 (endocrine extension)	DR

RS, recurrence score, LRR, locoregional recurrence; DR, distant recurrence

© 2023 Lee J et al. *JAMA Network Open*.

eTable 2. Univariate and multivariate analysis of risk factors for RFS in patients with low genomic risk

	Univariate		Multivariate	
	HR (95% CI)	*P* value	HR (95% CI)	*P* value
Menopausal statue				
Premenopausal	Ref.*			
Postmenopausal	0.84 (0.42–1.69)	0.63		
Tumor size (mm)				
≤20	Ref.			
>20	1.69 (0.92–3.13)	0.09		
Metastatic LN				
Negative	Ref.			
Positive	1.07 (0.54–2.15)	0.84		
PgR				
Negative	Ref.			
Positive	0.65 (0.26–1.66)	0.37		
ERBB2				
Negative	Ref.			
Low positive	0.98 (0.53–1.80)	0.94		
HG				
I or II	Ref.		Ref.	
III	5.04 (2.47–10.30)	<0.001	4.50 (2.15–9.40)	<0.001
LVI				
Negative	Ref.			
Positive	1.86 (0.99–3.52)	0.06		
Ki-67 (%)				
<20	Ref.		Ref.	
≥20	2.57 (1.39–4.74)	0.003	2.26 (1.19–4.31)	0.01
Breast surgery				
BCS	Ref.		Ref.	
Mastectomy	2.68 (1.45–4.94)	0.002	3.44 (0.84–14.09)	0.09
Chemotherapy				
Performed	Ref.			
Not performed	1.72 (0.67–4.42)	0.26		
OFS				
Not performed	Ref.			
Performed	1.10 (0.59–2.16)	0.79		
Radiotherapy				
Not performed	Ref.		Ref.	
Performed	0.39 (0.21–0.74)	0.003	1.06 (0.26–4.38)	0.939

© 2023 Lee J et al. *JAMA Network Open*.

*Reference value

RFS, recurrence-free survival; HR, hazard ratio; LN, lymph node; PgR, progesterone receptor; HG, histologic grade; LVI, lympho-vascular invasion; BCS, breast conserving surgery; OFS, ovarian function suppression

© 2023 Lee J et al. *JAMA Network Open*.

eTable 3. Comparison of baseline characteristics according to Ki-67 in patients with low genomic risk who did not receive chemotherapy

	All patients (N=1807), (%)	Patients with low Ki-67 (N=1262), (%)	Patients with high Ki-67 (N=545), (%)	*P* value
21-gene RS	14.6 ± 5.5	14.0 ± 5.3	15.8 ± 5.6	<0.001
Age (years)	49.9 ± 9.1	50.1 ± 8.7	49.5 ± 9.9	0.21
Menopausal status				0.85
Premenopausal	1183 (65.5)	828 (65.6)	355 (65.1)	
Postmenopausal	624 (34.5)	434 (34.4)	190 (34.9)	
Tumor size (mm)				<0.001
≤20	1212 (67.1)	886 (70.2)	326 (59.8)	
>20	595 (32.9)	376 (29.8)	219 (40.2)	
Metastatic LN				0.05
Negative	1461 (80.9)	1005 (79.6)	456 (83.7)	
Positive	346 (19.1)	257 (20.4)	89 (16.3)	
PgR				0.06
Negative	165 (9.1)	126 (10.0)	39 (7.2)	
Positive	1642 (90.9)	1136 (90.0)	506 (92.8)	
ERBB2				0.37
Negative	767 (42.4)	527 (41.8)	240 (44.0)	
Low positive	1040 (57.6)	735 (58.2)	305 (56.0)	
HG				<0.001
I or II	1704 (94.3)	1209 (95.8)	495 (90.8)	
III	98 (5.4)	48 (3.8)	50 (9.2)	
Unknown	5 (0.3)	5 (0.4)	0 (0.0)	
LVI				<0.001
Negative	1389 (76.9)	1,000 (79.2)	389 (71.4)	

© 2023 Lee J et al. *JAMA Network Open*.

Positive	418 (23.1)	262 (20.8)	156 (28.6)	
Breast surgery				0.05
BCS	1351 (74.8)	927 (73.5)	424 (77.8)	
Mastectomy	456 (25.2)	335 (26.5)	121 (22.2)	
Endocrine therapy				0.73
Not performed	16 (0.9)	10 (0.8)	6 (1.1)	
SERM	1222 (67.6)	848 (67.2)	374 (68.6)	
AI	568 (31.4)	403 (31.9)	165 (30.3)	
Others/Unknown	1 (0.1)	1 (0.1)	0 (0.0)	
OFS				<0.001
Not performed	1320 (73.0)	974 (77.2)	346 (63.5)	
Performed	487 (27.0)	288 (22.8)	199 (36.5)	
Radiotherapy				0.03
Not performed	424 (23.5)	314 (24.9)	110 (20.2)	
Performed	1383 (76.5)	948 (75.1)	435 (79.8)	

RS, recurrence score; LN, lymph node; PgR, progesterone receptor; HG, histologic grade; LVI lympho-vascular invasion; BCS breast conserving surgery; SERM, selective estrogen receptor modulator; AI, aromatase inhibitor; OFS, ovarian function suppression

© 2023 Lee J et al. *JAMA Network Open*.

eTable 4. Univariate and multivariate analysis of risk factors for RFS in patients with low genomic risk who did not receive chemotherapy

	Univariate		Multivariate	
	HR (95% CI)	*P* value	HR (95% CI)	*P* value
Menopausal status				
Premenopausal	Ref.*			
Postmenopausal	0.90 (0.43–1.88)	0.78		
Tumor size (mm)				
≤20	Ref.			
>20	1.33 (0.68–2.61)	0.41		
Metastatic LN				
Negative	Ref.			
Positive	1.36 (0.66–2.77)	0.41		
PgR				
Negative	Ref.			
Positive	0.52 (0.20–1.34)	0.18		
ERBB2				
Negative	Ref.			
Low positive	1.01 (0.52–1.97)	0.98		
HG				
I or II	Ref.		Ref.	
III	4.36 (1.90–10.01)	0.001	3.84 (1.66–8.90)	0.002
LVI				
Negative	Ref.			
Positive	1.77 (0.88–3.56)	0.11		
Ki-67 (%)				
<20	Ref.		Ref.	
≥20	2.39 (1.23–4.65)	0.01	2.51 (1.27–4.96)	0.008
Breast surgery				
BCS	Ref.		Ref.	
Mastectomy	3.16 (1.62–6.15)	0.001	2.01 (0.33–13.34)	0.43
OFS				
Not performed	Ref.			
Performed	1.24 (0.62–2.51)	0.54		
Radiotherapy				
Not performed	Ref.		Ref.	
Performed	0.32 (1.66–0.62)	0.001	0.55 (0.09–3.47)	0.52

*Reference value

RFS, recurrence-free survival; HR, hazard ratio; RS, recurrence score; LN, lymph node; PgR, progesterone receptor; HG, histologic grade; LVI, lympho-vascular invasion; BCS; breast conserving surgery; OFS, ovarian function suppression

© 2023 Lee J et al. *JAMA Network Open*.
