# Supplementary material for: Ki-67, 21-Gene Recurrence Score, Endocrine Resistance, and Survival in Patients With Breast Cancer
Source: JAMA Netw Open. 2023 Aug 30;6(8):e2330961. doi: 10.1001/jamanetworkopen.2023.30961 (PMC10469325; doi:10.1001/jamanetworkopen.2023.30961)
Supplement: Supplement 2. — Data Sharing Statement [file jamanetwopen-e2330961-s002.pdf]

Data Sharing Statement

Lee. Ki-67, 21-Gene Recurrence Score, Endocrine Resistance, and Survival in Patients With Breast Cancer. *JAMA Netw Open*. Published August 30, 2023.
doi:10.1001/jamanetworkopen.2023.30961

Data

Data available: Yes
Data types: Deidentified participant data, Participant data with identifiers, Data (not involving human participants), Data dictionary
How to access data: asg2004@yuhs.ac
When available: With publication

Supporting Documents

Document types: Informed consent form
How to access documents: asg2004@yuhs.ac
When available: With publication

Additional Information

Who can access the data: Anyone requesting the data
Types of analyses: For any purpose
Mechanisms of data availability: After approval of a proposal
